# Establishing Standardized Documentation for Anaphylaxis Treatment in a Tertiary Care Pediatric Allergy Clinic

**DOI:** 10.1097/pq9.0000000000000261

**Published:** 2020-02-15

**Authors:** Monica T. Kraft, Rebecca Scherzer, Kasey Strothman, Gayla Rogers, Tricia Montgomery, Mitchell H. Grayson

**Affiliations:** From the * Division of Allergy and Immunology, Department of Pediatrics, Nationwide Children’s Hospital, The Ohio State University College of Medicine, Columbus, Ohio; † Ohio State Wexner Medical Center, Columbus, Ohio.

## Abstract

**Introduction:**

Anaphylaxis is a potentially life-threatening allergic reaction. Common allergy clinic procedures, including oral food challenges and subcutaneous immunotherapy, carry a risk of anaphylaxis, the treatment for which is epinephrine. Our goal was to develop a standardized process for the management and documentation of allergic reactions that occur in a tertiary care pediatric allergy clinic.

**Methods:**

This was a single institution quality improvement pilot study. A multidisciplinary team from the allergy department designed, implemented, and studied the use of a standardized form for the documentation and treatment of allergic reactions within the clinic.

**Results:**

A standardized form was developed based on evidence-based guidelines for the management of allergic reactions and included space for documentation. Both clinic providers and staff approved the form. One year after the introduction, we reached 100% adherence for the use of the form in visits during which a patient experienced a severe allergic reaction requiring epinephrine. Two patients required transfer to the emergency room; the quality improvement form was utilized in these cases to document treatment and assist with the hand-off to emergency room personnel before transfer.

**Conclusions:**

We successfully implemented a standardized form for the treatment and documentation of anaphylaxis within our allergy clinic. The next steps focus on further integrating this form into the electronic medical record, determining compliance with evidence-based management of anaphylaxis, and formally assessing the use of the form as a handoff tool in the event of patient transfer.

## Abstract 2

Supplemental Digital Content is available in the text.

## INTRODUCTION

### Problem Description

Anaphylaxis is a potentially life-threatening allergic reaction that can include respiratory, cutaneous, gastrointestinal, and cardiovascular manifestations.^1,2^ In the allergy clinic, procedures such as oral food challenge (OFC) and subcutaneous immunotherapy (SCIT) are common but ultimately carry a risk of allergic reaction and anaphylaxis. An OFC is the gold standard for the diagnosis of food allergy. It is a procedure during which a patient consumes food or medication in gradually increasing doses in a supervised medical office setting to determine if it provokes an allergic reaction.^3^ SCIT or “allergy shots,” a common treatment for allergic rhinitis, asthma, and insect sting allergy, involves the subcutaneous injection of components of a patient’s known environmental allergens.^4^

Providers in the allergy clinic are prepared to treat allergic reactions triggered by OFC and SCIT. The pharmacologic treatment of anaphylaxis is the intramuscular injection of epinephrine. Providers may also administer oral antihistamines for adjunctive treatment or use in mild-moderate allergic reactions.^5,6^ Estimates of the incidence of systemic reactions, including anaphylaxis to SCIT, range from 1% to 4% of patients.^4^ For OFCs, prior reports included a rate of anaphylaxis requiring epinephrine between 2% and 3.9% of all challenges (11%–39.2% of failed challenges).^7–11^ Occasionally, those patients who need a higher level of medical intervention for their reactions may require transfer to the emergency department (ED) or admission to the hospital for further treatment and observation. Others have shown that guideline-based treatment of anaphylaxis during oral food challenges is variable—with some studies reporting the use of intramuscular epinephrine in all cases of severe anaphylaxis;^7,9^ whereas, another study found that in 5 children with signs of anaphylaxis during OFC for which treatment data were available, none received intramuscular epinephrine.^10^

In our Allergy and Immunology clinic at a tertiary care pediatric hospital, the number of OFC and SCIT visits continues to rise. Although rare, patients occasionally experience severe reactions requiring epinephrine and emergency transfer. Therefore, we wanted a system in place to standardize our approach to managing these reactions.

### Available Knowledge

The standard of care for the treatment of anaphylaxis is intramuscular epinephrine. However, the signs and symptoms of an allergic reaction can be subtle and variable depending upon the patient. Therefore, recognition and treatment of anaphylaxis can vary based on the provider and clinical situation. Little is known about the best way to ensure guideline-based treatment of allergic reactions and effective communication between healthcare providers for those patients who experience a reaction in the clinic. Standardized forms and checklists implemented in a variety of inpatient, outpatient, and surgical settings increase adherence to evidence-based guidelines, enhance communication between providers during hand-over, and improve written documentation of urgent or unexpected events.^12–14^

### Rationale

To our knowledge, there have been no prior studies on the use of a standardized form for the management of anaphylaxis in the allergy clinic or its possible benefit on transfers to the ED. To ensure appropriate documentation, communication, and management for these patients, we aimed to develop and implement a standardized form to outline evidence-based standard treatment of allergic reactions. Our goal was to have a visual reminder of the signs, symptoms, and treatment of anaphylaxis that would encourage providers to follow a standardized protocol in the event of a significant allergic reaction in the clinic. Furthermore, this form would allow for documentation of objective signs along with interventions for individual patients at the time of their reaction, rather than relying on provider recall for documentation after the event, and thus, would deliver a better record for the next provider at the time of transfer.

### Specific Aim

**Fig. 1.**
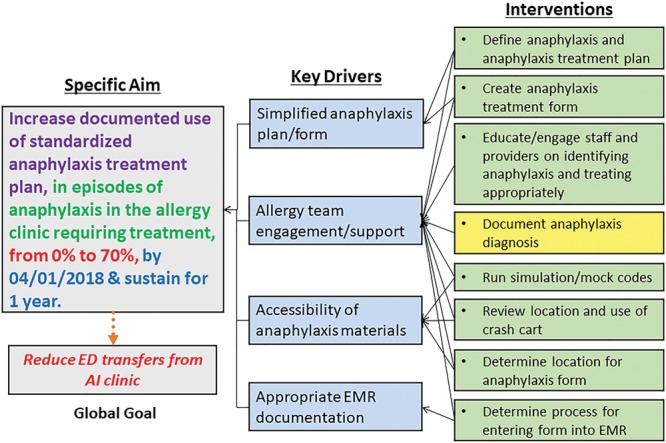
Key driver diagram. Our specific aim was to increase documentation of standardized anaphylaxis treatment during episodes of anaphylaxis in the allergy clinic. The ultimate goal remains that this intervention will hopefully lead to better in-clinic care, which will reduce the need for emergency transfers. Arrows show how specific interventions (on the right) apply to the key drivers (middle), which drive the specific aim and global goal. Colors of the interventions indicate completion (green) or currently being undertaken (yellow).

The specific aim for this project was to increase the documented use of a standardized anaphylaxis treatment plan during episodes of anaphylaxis in the allergy clinic requiring treatment, from 0% to 70%, and sustain its use in the clinic (Fig. 1). The global goal was to reduce the number of transfers to the ED from the allergy clinic.

## METHODS

### Context

This project was a quality improvement (QI) pilot study to design, implement, and utilize a standardized form for documentation of the treatment of severe allergic reactions, including anaphylaxis, in the allergy clinic. We created an interdisciplinary QI team composed of attending physicians, residents, nurses, and QI specialists from the Allergy and Immunology division with the charge of developing and implementing a standardized form for documentation of anaphylaxis treatment in the clinic.

### Interventions

Using the Institute for Healthcare Improvement QI principles, our team developed a key driver diagram (Fig. 1).^15^ The QI team developed the initial documentation form to incorporate the following components: (1) a place to document the timing of reaction symptoms and the treatment administered, and (2) a place to list the common symptoms, treatment, and clinic protocol for managing allergic reactions and anaphylaxis.

### Development of Standardized Form

The QI team modeled the initial standardized form after an existing document available in our clinic with which providers would be familiar. This form, the “food allergy action plan,” is provided to patients and families at their clinic visit. The form (see Supplemental Digital Content 1 at http://links.lww.com/PQ9/A159) describes symptoms and treatment options for acute allergic reactions in a clear, easy-to-follow diagram. On the reverse side, there are prompts for documenting the signs/symptoms of anaphylaxis and the timing of treatment administration. Focus groups of medical providers and clinic staff reviewed the form and made recommendations. Several revisions were made based on their critiques and suggestions. Examples of the important commentary from clinic staff included: use a large font, limit need for lengthy documentation by providing “checkbox” options, including documentation of patient disposition, and provide clear examples differentiating between mild allergic reactions and anaphylaxis.

### Implementation of the Standardized Form in Clinical Practice

Once finalized, we reviewed the form with all members of the clinic staff at a regularly scheduled division meeting before placing it in every clinic patient care room. We advised the nursing staff to start documentation using the form at the first sign of a patient’s allergic reaction. After the introduction, the clinic nurses suggested placing the forms on the back of each exam room door as a reminder and to make it easily accessible in the event of an allergic reaction. After the patient’s visit, the clinic staff scanned each form into the patient’s electronic medical record (EMR), a process used for all other paper documents from a patient’s office visit.

### Study of the Intervention

We utilized the pharmacy medication charge to identify all patients who received epinephrine in the allergy clinic. For this project, a severe reaction was defined as one requiring treatment with epinephrine and/or transfer to the ED. Through a retrospective chart review, we obtained the number of these patients who required transfer to the ED due to an allergic reaction for the periods before and after the intervention (implementation of a standardized form). Because of the relatively low frequency of emergency transfers (~2 per year), we collected data 1 year following intervention rather than continuously. The team also reviewed the number of severe allergic reactions in the allergy clinic in the year before and following the implementation of the form. As this was a new process, 0% of patients with an anaphylaxis event in the clinic had the standardized documentation in their EMR at baseline.

### Measures

The primary process measure evaluated was the utilization of the form for patients who experienced severe allergic reactions or anaphylaxis (administration of epinephrine) as identified by the presence of a scanned form in the patient’s EMR. A secondary outcome measure was appropriate completion of the form, including symptoms, medication administered and time of administration, vital signs, and disposition. Finally, we compared the overall rate of transfer from the allergy clinic with the ED in the year before the form’s introduction and the year following the intervention.

### Ethical Considerations

The Institutional Review Board at our institution approved the chart review of patients who had experienced an allergic reaction requiring treatment in our allergy clinic.

## RESULTS

**Fig. 2.**
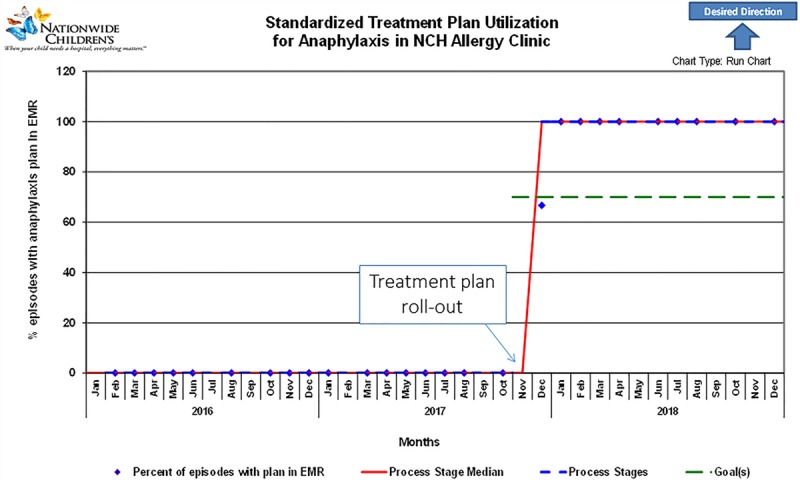
Run chart for implementation of the anaphylaxis treatment plan. The frequency per month that the EMR of a patient who received epinephrine included a copy of the completed anaphylaxis treatment plan (blue diamonds) increased quickly after implementation. Note that before this QI project (before November 2017), our clinic did not have a standardized form for the treatment of anaphylaxis. After developing the form with the input of medical providers and clinic staff, the standardized treatment plan was rolled-out in the clinic in November 2017. Redline represents the process stage median, and the green line indicates the goal (70%).

In the year before implementation of the standardized form (October 1, 2016, to October 31, 2017), 22 patients received epinephrine in the allergy clinic. Of these, 18% (4/22) required more than 1 dose of epinephrine, and 9% (2/22) required transfer to the ED. No patients required admission to the hospital. Following the introduction of the form (November 1, 2017, to November 30, 2018), 23 patients received epinephrine in the allergy clinic. Of these, 96% (22/23) had the standardized form scanned into the EMR (Fig. 2). The first eligible patient in the intervention period received epinephrine for anaphylaxis to immunotherapy and did not have the form completed; the subsequent 22 patient encounters did include this form, representing 100% compliance by the end of the study period. The encounter types with the completed form involved allergic reactions during 21 food challenge visits and 1 immunotherapy visit. Most patients (21/23) required only a single dose of epinephrine and were able to be discharged home directly from the clinic.

Our secondary outcome measure was the completion of documentation on the standardized form. Supplemental Digital Content 2 at http://links.lww.com/PQ9/A160 provides examples of completed forms. Of the forms completed during the study period, all included the name and time of medication administration, 1 or more of the patient’s symptoms, and at least 1 set of vital signs. The timing of the first entry on the form, however, was variable with some entries beginning at the first sign of clinical reaction (see Supplemental Digital Content 2B at http://links.lww.com/PQ9/A160). For others, the entry began at the time of medication (such as epinephrine) administration (see Supplemental Digital Content 2A at http://links.lww.com/PQ9/A160). This observation may indicate a lack of entry of early clinical signs and symptoms before the onset of anaphylaxis requiring treatment. Additionally, only 74% (17/23) of the forms included documentation of the patient’s disposition, time of clinic departure, and provider signature. It took a median of 20 days (range 5–43) from the time of the original patient visit to the scan of the completed form into the EMR.

The global aim was to reduce transfers from the allergy clinic to the ED. Two patients (9%) during the intervention period required 2 doses of epinephrine in the clinic and were subsequently transferred from the clinic to the ED with 1 patient requiring admission to the hospital. Although the same number of patients (2) were transferred in the 12 months before and 12 months after the intervention, the overall number of OFC visits continued to increase each year (from 360 OFCs in 2017 to 448 OFCs in 2018). Thus, the overall rate of transfer to the ED decreased. As the information on total OFCs was only available by the calendar year, an exact comparison of the change in the rate of transfer for the study period alone is not available. However, comparing overall transfer rates per year (rather than study period), we found that in the calendar year 2017, there were 360 OFCs and 3 ED transfers (0.83% per year) while for the calendar year 2018, there were 448 OFCs and 1 ED transfer (0.22% rate).

## DISCUSSION

### Summary

Patients undergoing common allergy clinic procedures such as oral food challenges and immunotherapy have a risk of experiencing allergic reactions in the allergy clinic. At our tertiary care allergy/immunology clinic, there are now over 500 patient visits for an OFC and over 400 SCIT patient visits yearly. A prior retrospective review from our center found an incidence of allergic reaction requiring epinephrine of 3.6% of OFC visits and 0.4% of SCIT visits.^16^ The demand for these visits continues to rise, and the number of transfers has the potential to increase. Before this project, we observed that in 5 years (January 1, 2012, to December 31, 2016), there were 871 in-office challenges, during which 2 patients required an emergency transfer from the allergy clinic for anaphylaxis (0.23%). In the following 6 months, from January to June of 2017, 2 out of 173 OFC patients (1.2%) were transferred to the ED. While all patients improved, and there were no adverse outcomes, there was no standardized process for the documentation surrounding the management of these patients. Additionally, documentation in the EMR of reactions and medication administration often occurs after the encounter and, thus, might not be available to the ED at the time of transfer. This increase in ED transfers in the first half of 2017 identified a need for standardization and documentation that we addressed through QI methodology.

The development of a standardized form documenting the presentation and management of anaphylaxis is important for both patient care and safety. The aim was to create this form to encourage clinic staff to follow evidence-based guidelines for the treatment of allergic reactions in the clinic, improve documentation during acute allergic reactions, and possibly reduce the need for transfer to the ED from the allergy clinic.

### Interpretation

Guidelines for the treatment of anaphylaxis recommend the use of intramuscular epinephrine.^1^ Despite the presence of these guidelines, other studies of allergic reactions during OFC have shown inconsistent adherence to these guidelines.^10^ While providers in our clinic are aware of evidence-based guidelines for the treatment of anaphylaxis, as the number of clinic procedures with the potential for an allergic reaction increased, we wanted to ensure standardization among providers of the protocol for the treatment of these reactions. Although there is limited allergy-specific data available for the best way to promote standardized treatment for anaphylaxis, shared experiences from other specialties in the hospital and clinic settings can be useful for encouraging adherence to guideline-based treatment. For example, a prior study demonstrated developing standardized bundles for treatment recommendations improves adherence to evidence-based guidelines for the management of *Staphylococcus aureus* bacteremia in admitted patients, with adherence to the bundle increasing by 43.8%.^13^ Additionally, in our intervention, we wanted to provide a physical form to accurately document the management of allergic reactions in real-time since clinic visit notes are often completed after the conclusion of the visit (and, thus, not available to the providers in the ED). While we did not examine provider notes, it is possible that just having the anaphylaxis form would improve the EMR documentation of the clinic visit. In fact, in a study of patients with shoulder dystocia, it was noted that a standardized form used in the delivery room improved the completeness of provider notes.^12^

In our study, we achieved 100% adherence to our documentation form shortly after introducing the form in the clinic. We attribute this success to the role the nursing staff played in form development and the placement of the forms in exam rooms. We also consider it a strength of our project that clinic providers used this form despite the relative infrequent (23 patients/year) episodes of severe allergic reactions requiring epinephrine use in the clinic. Only 1 patient who received epinephrine for a severe allergic reaction did not have the form completed. This patient was the first patient to receive epinephrine after implementation of the form, so the provider unfamiliarity with the new form may have contributed to a lack of use. It is also possible that the nurse or provider completed the form and then discarded it rather than scanning it into the patient’s EMR at the end of the visit. Additionally, this patient experienced a reaction after SCIT, whereas all patients who experienced anaphylaxis during an OFC had the form completed.

The high compliance with the use of this new documentation form suggests that it is easy to use during acute allergic reactions in the clinic. The most commonly omitted information on the form included the reason for visit, disposition, and provider signature, which may suggest that users of the form felt these were less important to document.

### Limitations

Limitations of this pilot study include a small sample size as the incidence of anaphylaxis in our allergy clinic is relatively low. Because of the low incidence, we reviewed records of patients who experienced anaphylaxis retrospectively 1 year following the intervention rather than in real-time. We attempted to mitigate the possibility of missed cases by using objective measures such as pharmacy charges for epinephrine use when identifying eligible patients. Epinephrine use would identify all potential patients transferred to the ED for an allergic reaction from our clinic (ie, no patients in the past 5 years have been transferred from the allergy clinic to the ED for a severe allergic reaction without having first received at least 1 dose of epinephrine). We confirmed in our review of the completed forms that all doses of medication (as ordered in the EMR during the visit) matched the hand-written form. However, the timing of symptom onset when compared with the initial time of documentation on the form is less easily identifiable. For instance, the first entry in some cases was with the first medication administered, and it is unclear whether there were earlier, undocumented symptoms present before the need for medical management. This observation represents an area for improvement: future iterations can study whether symptoms of anaphylaxis occurred before medication administration and be used to promote learning among providers and staff for early recognition of anaphylaxis. Additionally, for those patients transferred to the ED, we expected that a copy of the form would accompany the patient to the ED and help with the transition of care to the next provider; however, the utility of this form as a handoff tool is unclear based on documentation in the patient’s record. Finally, although we were primarily interested in the implementation of this standard form during severe allergic reactions, it is unclear how frequently or effectively this form is being used when patients experience milder allergic reactions in the clinic that do not require epinephrine.

### Next Steps

As we continue to revise and improve the process for standardizing the treatment and documentation for in-clinic allergic reactions, we plan to engage other providers in the review of this form. Future studies will include examining the utility of this form as a handoff tool for providers during transfers from the clinic to the ED. We are also actively working with providers to ensure episodes of anaphylaxis are documented adequately in the progress note and labeled as a “diagnosis” at that visit so that we can better track and monitor these episodes in the clinic. Additionally, we hope to assess whether the presence of an available protocol encourages evidence-based treatment for anaphylaxis in the clinic among providers. The next steps include the study of using this form in cases of milder allergic reactions not requiring epinephrine and review of whether there are delays between documentation of symptoms and administration of medication indicating divergence from guidelines and targets for future QI interventions.

Future iterations could include the incorporation of this form into an electronic format within the EMR itself to eliminate redundancies in charting and provide real-time documentation of identification and management of the allergic reaction in the patient visit record.

## CONCLUSION

We successfully achieved our goal of early implementation of a standardized form for the treatment and documentation of anaphylaxis in our allergy clinic. This on-going process will continue to address the growing need for standardized treatment, documentation, and communication for patients who experience allergic reactions in the clinic setting. The next steps include developing electronic systems to create more efficient documentation and facilitate patient handover in the event of transfer to the ED.

## DISCLOSURE

The authors have no financial interest to declare in relation to the content of this article. MHG has served on advisory boards for AstraZeneca, Genentech, Novartis, Genzyme, DBV Technologies, and Aimmune. All other authors have no conflicts to disclose.

## ACKNOWLEDGEMENTS

The authors wish to thank the other members of the team without whom this project could not have been implemented: Sean Gleeson, MD, Susan Savola, RN, Sabrina Helwagen, RN, and Anna Wells, RN. This project was developed as part of MHG’s participation in the Nationwide Children’s Hospital Quality Improvement Essentials course.

## Supplementary Material


