# Supplementary material for: Establishing Standardized Documentation for Anaphylaxis Treatment in a Tertiary Care Pediatric Allergy Clinic
Source: Pediatr Qual Saf. 2020 Feb 15;5(2):e261. doi: 10.1097/pq9.0000000000000261 (PMC7190245; doi:10.1097/pq9.0000000000000261)
Supplement: Supplementary file 1 [file pqs-5-e261-s001.pdf]

NATIONWIDE CHILDREN'S
When your child needs a hospital, everything matters.℠

PATIENT IDENTIFICATION

PROTOCOL FOR IN-CLINIC ALLERGIC REACTIONS

This protocol has a front and back page and should be used at the first sign of an allergic reaction. Please document vital signs, concerning physical exam findings and any medications administered in the table below with corresponding time. Turn this page over to review the protocol for management of an acute reaction.

Patient presents for (circle one): **In-Office Challenge** **Immunotherapy** **Other**

Patient Weight ________________ **Appointment/ Start Time_______________**

Time	Physical Exam (Check any findings present, specify location)		Vital Signs			Drug	Dose/ Route
	□ Hives<br>□ Wheeze<br>□ Rhinitis/ Sneeze<br>□ Cough	□ Angioedema<br>□ Emesis<br>□ Other ___________	HR	BP ____ / _____	RR/ O2 Sat	□ Epinephrine<br>□ Cetirizine<br>□ Diphenhydramine<br>□ Albuterol<br>□ ______________	
	□ Hives<br>□ Wheeze<br>□ Rhinitis/ Sneeze<br>□ Cough	□ Angioedema<br>□ Emesis<br>□ Other ___________	HR	BP ____ / _____	RR/ O2 Sat	□ Epinephrine<br>□ Cetirizine<br>□ Diphenhydramine<br>□ Albuterol<br>□ ______________	
	□ Hives<br>□ Wheeze<br>□ Rhinitis/ Sneeze<br>□ Cough	□ Angioedema<br>□ Emesis<br>□ Other ___________	HR	BP ____ / _____	RR/ O2 Sat	□ Epinephrine<br>□ Cetirizine<br>□ Diphenhydramine<br>□ Albuterol<br>□ ______________	
	□ Hives<br>□ Wheeze<br>□ Rhinitis/ Sneeze<br>□ Cough	□ Angioedema<br>□ Emesis<br>□ Other ___________	HR	BP ____ / _____	RR/ O2 Sat	□ Epinephrine<br>□ Cetirizine<br>□ Diphenhydramine<br>□ Albuterol<br>□ ______________	
	□ Hives<br>□ Wheeze<br>□ Rhinitis/ Sneeze<br>□ Cough	□ Angioedema<br>□ Emesis<br>□ Other ___________	HR	BP ____ / _____	RR/ O2 Sat	□ Epinephrine<br>□ Cetirizine<br>□ Diphenhydramine<br>□ Albuterol<br>□ ______________	
	□ Hives<br>□ Wheeze<br>□ Rhinitis/ Sneeze<br>□ Cough	□ Angioedema<br>□ Emesis<br>□ Other ___________	HR	BP ____ / _____	RR/ O2 Sat	□ Epinephrine<br>□ Cetirizine<br>□ Diphenhydramine<br>□ Albuterol<br>□ ______________	

Time of Discharge _____________ **RN Signature ___________________Date/Time__________**

Practitioner Signature ___________________ Date/Time__________

This form represents a general guideline only and is subject to the clinical judgement of the provider. All dosing for medications administered should be determined by the ordering provider.

Last Updated 9/19/2017

PATIENT IDENTIFICATION

FOR MILD OR LOCAL ALLERGIC REACTION[1]

Vitals → **Antihistamine** → **Discharge**

- **Vitals:** Record time and VS at first sign of reaction. Notify Provider
- **Antihistamine:** Consider Cetirizine (Zyrtec) or Diphenhydramine (Benadryl)
- **Discharge:** If no additional symptoms after 30-60 mins, patient is ready for discharge

[1]**Signs of Mild reaction include:**
- Hives on face or body
- Itchy mouth
- Rhinorrhea, sneezing
- Mild nausea/ discomfort

FOR SEVERE ALLERGIC REACTIONS[2]

[2] **Signs of anaphylaxis or severe reaction include any one or more of the following body system involvements:**

Lung: Shortness of breath, wheeze, repetitive cough
Throat: Tightness, closing or fullness, trouble breathing or swallowing
Heart: Pale, blue, faint, weak pulse, light-headedness, passing out
Mouth: Swelling of tongue
Gut: Repetitive or severe vomiting, diarrhea, abdominal pain
Skin: Severe swelling

Weight	Epinephrine Dose
<30 kg	0.15 mg IM (i.e. Epi Pen Jr)
≥ 30 kg	0.3 mg IM (i.e. Epi Pen Auto-Injector)

ANAPHYLAXIS or SEVERE REACTION[2]

Vitals | Notify MD

↓

EPINEPHRINE IM (#1)

Record Time | Monitor closely

↓ After 5 minutes

Symptoms resolve

↓

OBSERVE (MINIMUM 1 HOUR)

Monitor for new or progressing symptoms | Vitals

↓ 1 hour

DISCHARGE

Record Vital Signs, Notify Provider | Scan front page into patient's chart

Symptoms remain or worsen

↓

REPEAT **EPINEPHRINE** (#2)

Record Time And Vitals | Monitor for signs of clinical instability

↓ If Unstable

CALL CODE BLUE x22233

Stay with patient until code team arrives and provide copy of front page | **Keep** front copy to scan into patient's chart

Continue to repeat IM Epi as needed

This form represents a general guideline only and is subject to the clinical judgement of the provider. All dosing for medications administered should be determined by the ordering provider. *Last Updated 9/19/2017*
